# Left atrial volume by cardiac CTA prior to catheter ablation: comparison to echocardiography and association with recurrent atrial fibrillation

**DOI:** 10.1186/s13104-023-06313-2

**Published:** 2023-04-07

**Authors:** Wissam Alajaji, Ottorino Costantini, Tyler L. Taigen, Mark A. Iler

**Affiliations:** 1 Summa Health Heart and Vascular Institute, 95 Arch St, Suite 300, Akron, OH USA; 2 Northeast Ohio Medical University, Akron, OH USA; 3 Cleveland Clinic Heart and Vascular Institute, Cleveland, OH USA; 4 Lerner College of Medicine of Case Western Reserve, Cleveland, OH USA

**Keywords:** Left atrial volume, Cardiac CTA, Echocardiography, Atrial fibrillation, Pulmonary vein isolation

## Abstract

**Objective:**

Left atrial volume index (LAVI) by echocardiography can be challenging and its accuracy is highly dependent on image quality. Cardiac computed tomography angiography (CTA) can overcome echocardiographic LAVI measurement challenges; however, data are limited. Therefore, we evaluated the reproducibility of LAVI by CTA, its correlation to echocardiography, and its association with recurrence of atrial fibrillation (AF) after pulmonary vein isolation (PVI) in this retrospective cohort study of patients who underwent CTA prior to PVI. LAVI was measured by CTA and echocardiography using the area length method.

**Results:**

74 patients had echocardiography and CTA within 6 months and were included in this study. The interobserver variability of LAVI measured by CTA was low (1.2%). CTA correlated with echocardiography but found larger LAVI values by a factor of 1.6. Also, LAVI cut off of ≥ 55 ml/m^2^ measured by CTA correlated with recurrent AF after PVI (adjusted Odds Ratio 3.47, p = 0.033).

## Introduction

Left atrial volume index (LAVI) has been shown to correlate with cardiovascular outcomes in atrial fibrillation (AF) and procedural success after pulmonary vein isolation (PVI) [1–4]. Echocardiography is the most widely used test for measurement of LAVI. Currently, LAVI is the preferred measurement to estimate left atrial (LA) size over other techniques such as anteroposterior diameter and single plane area by planimetry [5]. However, LAVI measured by echocardiography has the usual inherent limitations of planar imaging and dependence on quality of acoustic windows.

Owing to its accuracy in delineating 3-dimensional (3D) cardiac structure, computed tomography angiography (CTA) is commonly used prior to PVI procedures for LA and pulmonary vein anatomy assessment as well as ablation approach planning. CTA can easily evaluate LAVI. There are few published studies which evaluated LAVI measurements by CTA; however, LAVI measurements were mostly obtained by automated methods using endocardial edge detection which remain inconsistent for excluding the pulmonary veins, left atrial appendage, and mitral annular plane. Furthermore, only very few studies have compared the volumes obtained by CTA with those by echocardiography or correlated those volumes to outcomes [6–8].

Therefore, we sought to study a simple method of measuring LAVI by CTA using an area-length method which is the recommended and most used technique in echocardiography. The aims of this study were to (1) evaluate the reproducibility of CTA LAVI measurements, (2) compare CTA LAVI correlation to echocardiographic LAVI, and (3) to compare how the two techniques correlate with recurrence of AF after PVI.

## Main text

### Materials and methods

#### Study design

This is a retrospective cohort study of patients who underwent CTA prior to PVI between January 2013 and June 2016. The protocol was approved by the institutional review board at Summa Health (Akron, Ohio). Consecutive patients who underwent CTA prior to PVI and echocardiography within 6 months of the cardiac CTA were included.

First, CTA LAVI measured by two blinded observers was evaluated for inter-observer agreement. Second, CTA LAVI was compared with the corresponding echocardiographic LAVI. Finally, the relationship of CTA LAVI and echocardiographic LAVI to risk of AF recurrence was analyzed.

#### Echocardiographic examination

**Fig. 1:**
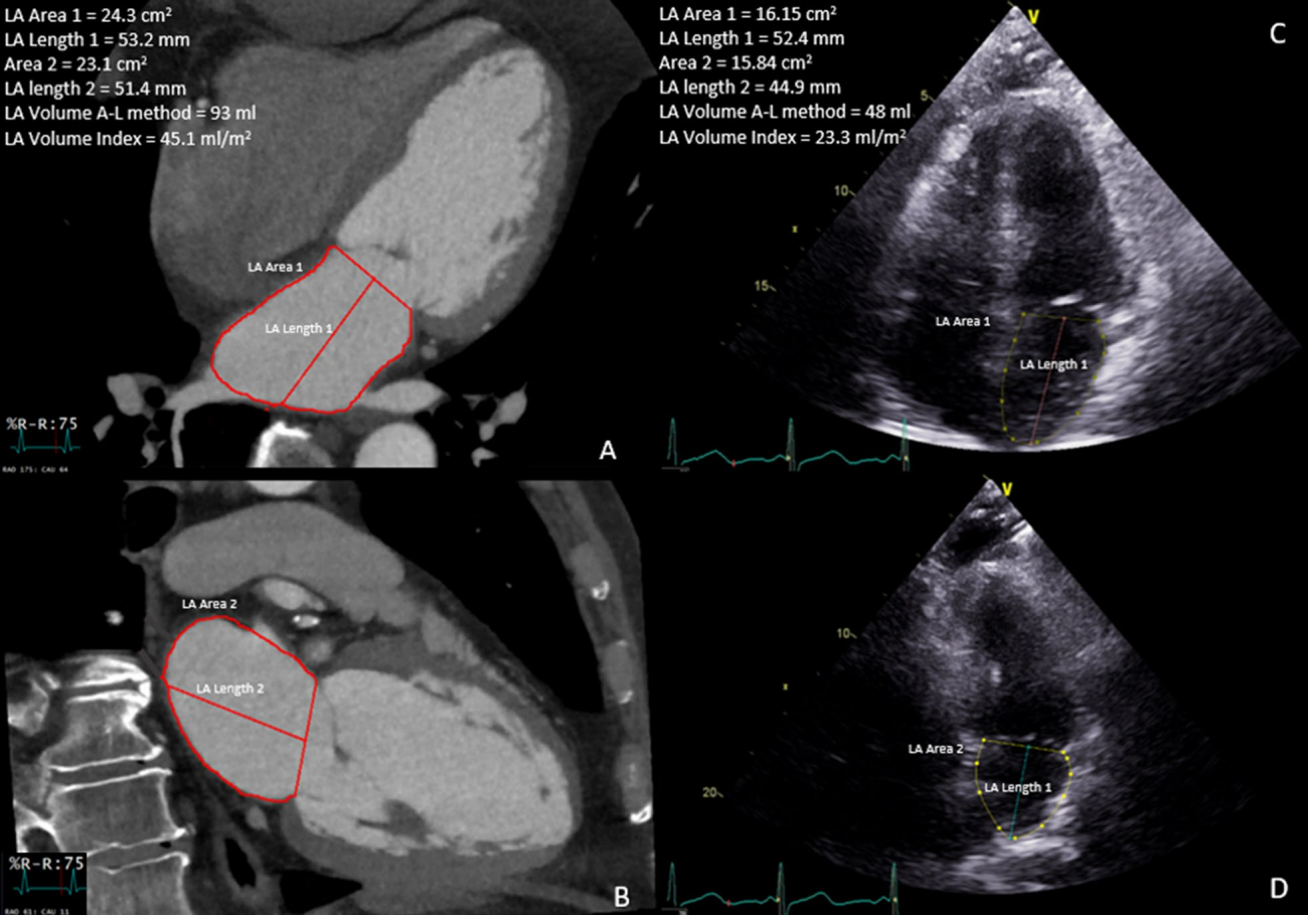
Comparison of computed tomography and echocardiography LA volume measurements obtained from the same patient with atrial fibrillation undergoing evaluation for pulmonary vein isolation procedure. **A**, Computed tomographic 4-chamber reconstruction at the maximal LA basal and longitudinal dimensions. **B**, Computed tomographic 2-chamber reconstruction at the maximal LA basal and longitudinal dimensions. A and B show LA volume measurement by the CT area-length method. **C**, Echocardiographic 4-chamber view. **D**, Echocardiographic 2-chamber view. C and D show LA volume measurement by echocardiographic area-length method. The inherent challenges of echocardiography, including adequate visualization of the LA, foreshortening and tracing difficulties are demonstrated

All echocardiograms at our institution are performed in an accredited clinical laboratory and interpreted by board certified echocardiographers. Left atrial diameter, area, and volume measurements are performed according to American Society of Echocardiography recommendations. In the parasternal long axis view, left atrial diameter is measured at end systole from the level of the aortic annulus to the posterior left atrial wall. In optimized apical 4 and 2 chamber views, left atrial area at end systole is measured by planimetry with careful exclusion of the appendage and pulmonary veins (Fig. 1). Left atrial length is measured as the perpendicular distance from the middle of the plane of the mitral annulus to the superior aspect of the LA. LAVI is calculated using the previously described biplane area length method:

$$LAVI \, = \, \left[ {\left( {0.85} \right) \, * \, LA \, Area\,in \, 4 \, chamber \, view \, * \, LA \, Area\,in \, 2 \, chamber \, view/LA \, length} \right]/Body \, Surface \, Area$$

#### Cardiac CTA examination

All patients underwent cardiac CTA using a 640 slice CT scanner with prospective gating. To limit motion artifacts, images were obtained during diastole, and reconstructed in diastasis (~ 70 to 75% of the RR interval). CTA LAVI measurement was performed on commercially available postprocessing software (iNtuition, TeraRecon, Inc., Foster City, CA) according to the following method analogous to echocardiographic LAVI. Optimal 4 and 2 chamber multiplanar reconstructions were created using a line from the left ventricular apex to the posterior LA wall, bisecting the mitral annulus. Multiple orthogonal views were compared to carefully avoid foreshortening of the LA and to exclude the aortic root if possible (Fig. 1). Area was measured by planimetry in both views based on a straight line at the mitral annulus and excluding the LA appendage and pulmonary veins at their ostia. The length was measured from the middle of the mitral annular line to the middle of the posterior wall. The shorter of the two lengths was used to calculate LAVI using the same formula used for echocardiographic LAVI. All CTA LAVI measurements were performed independently by two physicians (WA and MI) who were blinded to all other echocardiography and CTA measurements and patient outcomes.

#### Statistical analysis

Intra-class correlation for CTA LAVI was calculated using the absolute agreement of average measures, with reliable CTA LAVI measurements defined as intra-class correlation ≥ 0.75. Bland–Altman analysis was used to determine the concordance of CTA and echocardiography for LAVI. Receiver operator characteristics curves were determined for CTA LAVI and echocardiographic LAVI for association with AF recurrence. Using the maximum of Youden’s J index, the optimal cut off values for CTA LAVI and echocardiographic LAVI were identified. Chi Square test was performed to determine the significance of cutoff values in relationship to AF recurrence. All statistical tests were performed using SPSSv25.0 software (IBM Corp., Armonk, NY). Two-sided p < 0.05 was considered statistically significant.

## Results

### Patients

**Fig. 2:**
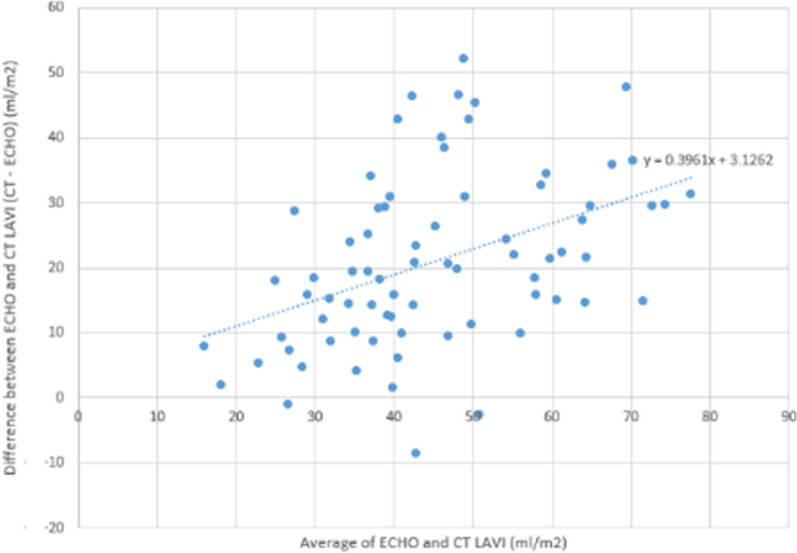
Bland Altman Plot for echocardiography and CCTA values. CCTA LAVI is systematically greater than echocardiographic LAVI (p < 0.001) via paired samples t test. The slope is significant (p < 0.001) indicating a greater divergence with larger LAVI values

**Fig. 3:**
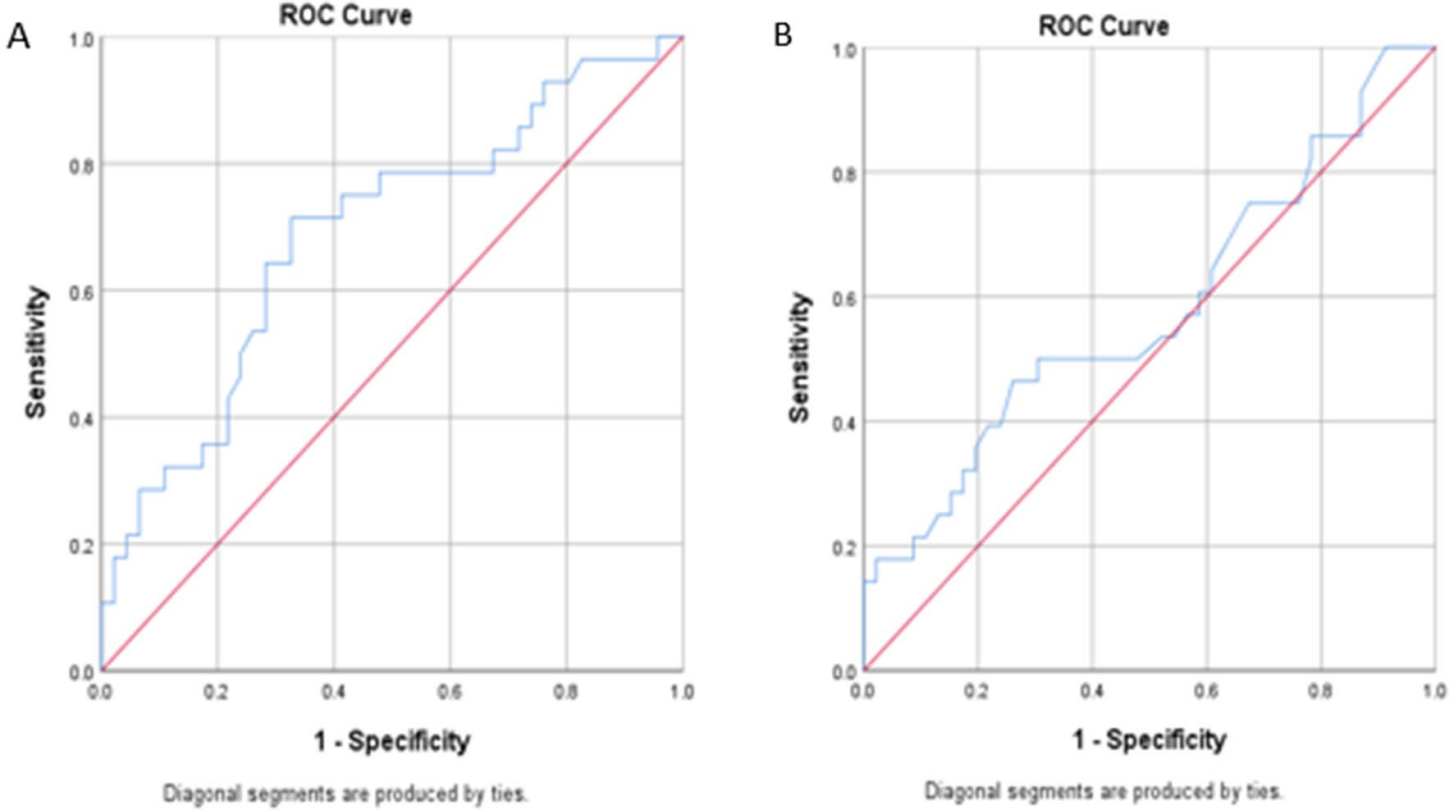
Area under the curve values for CCTA LAVI ≥ 55 ml/m2 and echocardiographic LAVI ≥ 34 ml/m2. **A**, CCTA LAVI ≥ 55 ml/m2, AUC 0.689 ± 0.065 (95% CI 0.563–0.816), P = 0.007 and sensitivity = 71%, 1-Specificity = 33%. **B**, Echocardiographic LAVI ≥ 34 ml/m2, AUC 0.579 ± 0.071 (95% CI 0.440–0.718), P = 0.256

404 patients underwent PVI at Summa Akron City Hospital between June 2013 and June 2016. Of those, 74 had both CTA and echocardiography performed within 6 months of the index procedure and were included. Of the 74, 45 (61%) patients had persistent and 29 (39%) had paroxysmal AF as defined by the 2014 multi-society guidelines [9]. Patient characteristics are listed in Table 1. Group A are patients without AF recurrence (n = 46) and Group B are patient with AF recurrence (n = 28). Patients with AF recurrence were more likely to have persistent AF than paroxysmal AF at baseline (75% versus 25%) compared to those without recurrence (52.2% vs 47.8%), p = 0.051. The interobserver variability was very low (1.2%) between the two blinded CTA readers consistent with high reproducibility. The interclass correlation (ICC) of LAVI by CTA is 0.988 (0.981–0.993). Also, CTA and echocardiographic LAVI measurements correlated with a correlation coefficient between 0.3961, P value < 0.001 (Fig. 2). Last, The sensitivity, specificity and area under the curve for AF recurrence after PVI using CTA LAVI of ≥ 55 ml/m^2^, were 71%, 67%, and 0.689, respectively (Fig. 3). Whereas the echocardiographic LAVI cutoff value ≥ 34 ml/m^2^ [5] demonstrated no correlation with AF recurrence after PVI (area under the curve 0.579, P = 0.256) (Fig. 3).

**Table 1:** Patient Characteristics and LA Size stratified by AF Recurrence

Variable	Patient Study cohort		P-value
	No AF recurrence after PVI	AF recurrence after PVI	
Number, n	46	28	
Age mean (SD), year	62.9 (11.26)	68.2 (8.83)	0.084
Gender—n (%)			0.370
Male	32 (69.6)	16 (59.3)	
Female	14 (30.4)	11 (40.7)	
Body mass index mean (SD), kg/m2	30.0 (4.23)	29.2 (5.22)	0.229
Ejection fraction mean (SD), %	56.3 (8.62)	54.5 (13.19)	0.611
CHADS VASc score—n (%)			0.721
0	5 (10.9)	1 (3.7)	
1	6 (13.0)	7 (25.9)	
2	16 (34.8)	5 (18.5)	
3	6 (13.0)	6 (22.2)	
4	10 (21.7)	6 (22.2)	
5	3 (6.5)	1 (3.7)	
6	0	1 (3.7)	
AF Type—n (%)			0.051
Paroxysmal	22 (47.8)	7 (25.0)	
Persistent	24 (52.2)	21 (75.0)	
AF duration mean (SD), months	20.1 (36.91)	27.0 (30.67)	0.058
Accessory pulmonary vein—n (%)	5 (10.9)	7 (25.0)	0.192
Common pulmonary vein antrum, n (%)	10 (21.7)	2 (7.1)	0.118
Separate ostia pulmonary veins, n (%)	3 (6.5)	2 (7.1)	1.000
Prior pulmonary vein isolation, n (%)	2 (4.3)	1 (3.6)	1.000
LA volume by CT, mean (SD), mL	103.7 (32.77)	129.2 (37.96)	0.004
LAVI by CT mean, (SD), mL/m2	51.0 (16.57)	63.4 (18.16)	0.007
LA Diameter by echocardiography, mean (SD), cm	4.13 (0.64)	4.20 (0.63)	0.872
LA Area by echocardiography MEAN (SD), cm2	20.8 (5.50)	22.7 (5.30)	0.175
LA volume by echocardiography, Mean (SD), mL	66.8 (23.54)	76.4 (28.95)	0.174
LAVI by echocardiography, mean (SD), mL/m2	32.8 (11.67)	37.7 (14.41)	0.256

### Analysis

#### Correlation between CTA LAVI and echocardiographic LAVI

CTA LAVI correlated with echocardiographic LAVI and yielded larger volumes. Using the correlation equation: y = 0.3961x + 3.1262, CTA LAVI was found to be 1.6*echocardiographic LAVI (Fig. 2). Thus, the CTA values that correlate to the American Society of Echocardiography guideline [5] LAVI cutoff values for mild (34 to 41 ml/m2), moderate (42 to 47 ml/m2) and severe (≥ 48 ml/m2) LA enlargement are 55 to 65 ml/m2, 66 to 76 ml/m2, ≥ 77 ml/m2, respectively [5]. However, at higher LAVI values, the echocardiography and CTA curves diverge, suggesting less correlation.

#### CTA LAVI Compared to Echocardiographic LAVI to Predict AF Recurrence

Patients were divided into two groups based on AF recurrence after PVI during a mean follow-up period of 24 months (range, 6 to 72 months). Group A is patients without AF recurrence and group B is patients with AF recurrence. The average time to first AF recurrence was 8.8 months. There was no difference in AF recurrence based on echocardiographic LA diameter, LA area, or LAVI. The echocardiographic LAVI cutoff value ≥ 34 ml/m^2^ [5] demonstrated no correlation with AF recurrence after PVI (area under the curve 0.579, P = 0.256) (Fig. 3). In contrast, CTA LAVI was significantly larger in those patients with subsequent AF recurrence (63.4 ± 18.16 compared to those without AF recurrence 51.0 ± 16.57 ml/m2, P = 0.007, Table 1). The adjusted odds ratio for AF recurrence using a CTA LAVI cutoff value of ≥ 55 ml/m^2^ was statistically significant (OR 3.47, P = 0.033, Table 2). The sensitivity, specificity and area under the curve for AF recurrence after PVI using CTA LAVI of ≥ 55 ml/m^2^, were 71%, 67%, and 0.689, respectively (Fig. 3).

**Table 2:** Logistic Regression for AF Recurrence Using CCTA LAVI

Variable	Full model	P-value	Reduced model	P-value
	Adjusted odds ratio		Adjusted odds ratio	
Age, years	1.031	0.346	N/S	N/S
AF duration, months	2.057	0.232	N/S	N/S
Persistent AF	1.009	0.251	N/S	N/S
CT LAVI ≥ 55 ml/m2	3.47	0.033	4.908	0.002

p < 0.10 required for initial model and final model inclusion

Multivariate analysis was performed in a logistic regression model, including CTA LAVI, age, duration of AF, and pattern of AF. Of these factors, only CTA LAVI was predictive of AF recurrence.

## Discussion

**Fig. 4:**
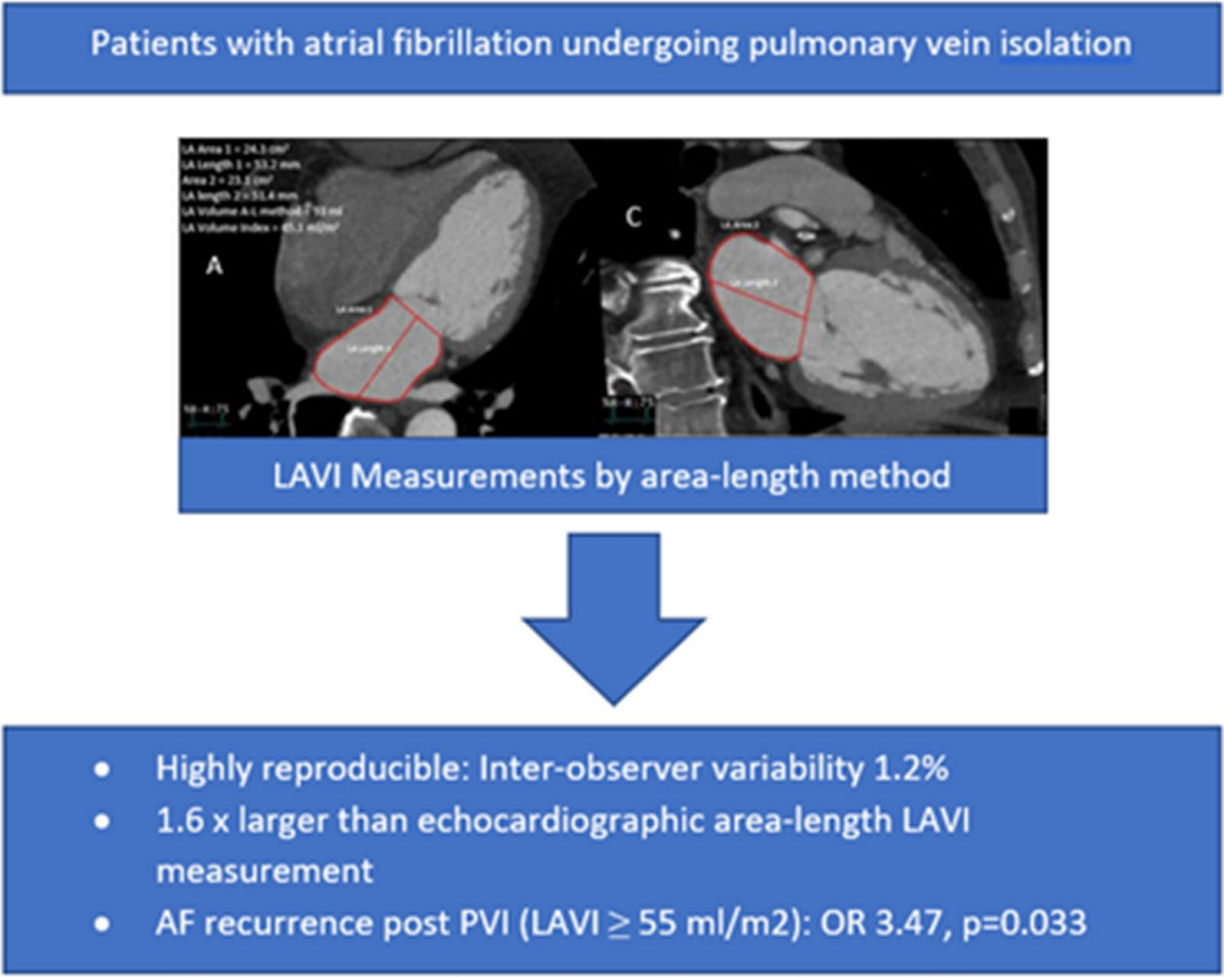
Study summary. This figure highlights area-length method used during this study for LAVI measurements in patients with AF prior to undergoing PVI and summarizes key findings

This study (Fig. 4) demonstrates that CTA LAVI is highly reproducible and correlates well with echocardiographic LAVI but consistently results in a larger LA volume by a factor of 1.6. In addition, this study suggests that CTA LAVI may be more useful than echocardiographic LAVI to predict AF recurrence. Also, the area-length method for LAVI measurement by CT is technically quick, easy to perform and may be attractive to use in future studies that involve LAVI measurements. However, as 3D volume rendering becomes more available and reliable, it may be more favorable to use direct 3D derived LAVI over area-length method derived LAVI.

### CTA LAVI reproducibility

The inter-observer variability of only 1.2% demonstrates that using a standardized approach, the CTA LAVI method is highly reproducible. This is likely due to the inherent advantages of CT and post-processing software for optimization of standardized imaging planes.

Compared to 2D echocardiographic LAVI, 3D echocardiography and CTA seem to have much lower inter-observer variability. King et al. reported interobserver variability rates of 14.6% and 5% for echocardiographic LAVI and 3D echocardiographic volume, respectively [10]. The higher variability using echocardiography can be explained by its inherent dependence on adequate acoustic windows and selection of correct imaging parameters. CTA does not have these limitations. Thus, a reproducible technique to measure LAVI by CTA may improve the power to predict important outcomes.

### Correlation between CTA LAVI and echocardiographic LAVI

This study revealed that CTA LAVI at mid-diastole correlates well with echocardiographic LAVI at end-systole but yields consistently larger values by about 60%. This is consistent with prior published data [11]. The difference between CTA and echocardiography can be explained by significant underestimation of LA volume by the latter, especially using 2D techniques [12]. As the longitudinal axes of the LV and LA frequently lie in different planes, foreshortening of the LA by echocardiography is common. Taken together with inferior spatial resolution, echocardiography provides consistently underestimated and less reproducible volumes. This may limit its prognostic utility for clinical and investigative purposes.

### CTA LAVI and AF recurrence

CTA LAVI correlated with AF recurrence after PVI, whereas echocardiographic LAVI did not. The association between a larger CTA LAVI and AF recurrence is expected on the basis of more advanced atrial remodeling and fibrosis. Using the guideline-based cutoff value of ≥ 34 ml/m^2^ for LA enlargement [5], echocardiographic LAVI did not predict AF recurrence after PVI. However, its counterpart, CTA LAVI ≥ 55 ml/m2, was associated with AF recurrence following PVI. Interestingly, out of the 28 patients with AF recurrence, 20 had LAVI ≥ 55 ml/m2 of which 19 were persistent AF and 1 paroxysmal. For the remaining 8 with AF recurrence and LAVI < 55 ml/m^2^, 5 had paroxysmal AF and 3 had persistent AF. Therefore, the combination of LAVI ≥ 55 and persistent AF may indicate a higher risk group for recurrence.

The relationship between LA volume and AF outcomes is likely to be continuous. The present study calls into question the utility of the guideline-based cutoff of echocardiographic LAVI ≥ 34 ml/m^2^ [5] as a predictor of AF recurrence, and suggests that CTA LAVI may be better for this purpose because it more accurately measures true LA volume. It is also possible that echocardiographic LAVI is useful for prognostication only at larger values. We acknowledge that the present study was underpowered to demonstrate a smaller but true effect size for echocardiographic measurements.

## Conclusion

Measurement of LA volume by CTA using a standardized area-length method is highly reproducible and results in consistently greater LA volume than echocardiography. Furthermore, CTA LAVI predicts AF recurrence in patients undergoing PVI better than echocardiographic LAVI and warrants further study.

## Study limitations

The primary study limitations are those of all observational studies. Care should be taken in interpreting the finding that CTA LAVI is predictive of AF recurrence. Study size and duration of follow up were relatively small. These findings should be considered hypothesis-generating and prospective studies of CTA LAVI as a predictor of AF outcomes should be undertaken.

CTA image acquisition occurred in mid-diastole rather than at end systole, the usual timing for LA measurements when volumes are largest. This suggests the difference between CTA and echocardiography is even greater than suggested. Arguably, precise acquisition and selection of the correct end systolic frame may be less reproducible than during diastasis, when image quality is optimized for structural evaluation. We believe that measuring CTA LAVI at end-systole would unlikely change the results significantly, and if so would only accentuate the measured difference.

Furthermore, automated segmentation software was not used to measure CTA LAVI. This technique has improved and become more widely used since the time of our study.

## Data Availability

Not available.

## Abbreviations

LAVI: Left atrial volume index
CTA: Computed tomography angiography
AF: Atrial fibrillation
PVI: Pulmonary vein isolation
LA: Left atrial
